# Magnetically Decoupled Janus Bound States in the Continuum for Chirality–Topology Control

**DOI:** 10.1002/nap2.70134

**Published:** 2026-05-27

**Authors:** Yadong Yue, Du Li, Kun Liang, Li Yu

**Affiliations:** ^1^ State Key Laboratory of Information Photonics and Optical Communications School of Physical Science and Technology Beijing University of Posts and Telecommunications Beijing China

**Keywords:** bound states in continuum, magneto‐optical materials, photonic crystals

## Abstract

Bound states in the continuum (BICs) with infinite *Q* factors and topological polarization charges have emerged as a pivotal platform in quantum information. The Janus BICs carrying asymmetric topological charges exhibit great potential for nonreciprocal photonics devices. Here, the topological properties of the Janus BICs are preserved while decoupled chirality is induced, which enhances the system's advantages by providing an extra degree of freedom. In contrast to previous Janus BICs, where chirality is coupled to topological charges, we achieve decoupled control through time‐reversal symmetry breaking. The theory of modified nonreciprocal temporal coupled mode theory (TCMT) with perturbation is developed to describe our system, which consists of the bi‐layer photonic crystals (PhCs) with $C_{3v}$ symmetry and magneto‐optical (MO) $C_{6v}$ symmetry slabs. When time‐reversal symmetry is broken, the degenerate BICs in the MO slab split into opposite chiral modes, whose chiralities are controllable through the direction and strength of the external magnetic field. By combining the simulation method, we observe the independent modulation of topological charges and chiralities in the polarization of the far field and evolution of C points with the change of separation. Our findings open pathways for applications in chirality‐dependent devices and asymmetric light–matter interaction.

## Graphical abstract

This article demonstrates the Janus BICs, which are bi‐layer photonic crystal (PhCs) structures composed of C_3v_ and magneto‐optical C_6v_ symmetry slabs. It offers a unique platform for independent controlling chirality and topological charge, which successfully realizes the decoupling of topological charge and chirality. Our work advances the field of topological photonics by introducing a new avenue for manipulating phase singularities to achieve strong chirality with robust topological protection.

## 1 Introduction

Topological defects have recently garnered significant attention in photonics, particularly through manifestations such as bound states in the continuum (BICs) [1, 2, 3, 4, 5, 6] and unidirectional guided resonances [7]. These defects carry nonzero topological charges, defined by the winding number of the surrounding polarization states, and manifest as polarization vortices in momentum space [6, 8]. The high‐Q factors and topological polarization configurations of BICs make them suitable for a wide range of applications: lasing [9], spin–orbit interaction [10], sensing [11], Bose–Einstein condensation [12], vortex beam generation [9, 13], and exciton polaritons [14], whereas unidirectional guided resonances with topological charges that differ on the upward and downward sides exhibit unidirectional radiation [15]. C points, which possess large chirality with half‐integer topological charges [16], provide new avenues for chiral optics. The manipulation of phase singularities in the generation of optical vortices is significant in various fields, not only optical communications but also quantum information processing [17]. The singularity in momentum space created by the spin–orbit interaction will lead to promising applications in vortex beam generation [13] and beam shift [10, 18]. Enhancing chirality is a significant goal for applications of the chiral light–matter interaction [19]. C points show large chirality, but their emergence usually deviates from the normal direction [20]. The application requires intrinsic chirality, which motivates the design of chiral quasi‐BICs that exhibit both strong intrinsic chirality and high‐Q factors [21, 22, 23].

New topological singularities, such as Janus BICs, which exhibit different topological charges on the two sides, introduce novel optical properties and functionalities [24]. Out‐of‐plane asymmetric polarization vortices make Janus BICs a promising application in nonlinearity‐induced nonreciprocal devices [25, 26]. However, in existing Janus BICs, chirality and topological charge remain coupled, limiting independent control and restricting flexibility in device design. Magneto‐optical (MO) materials, whose optical properties can be modulated by external magnetic fields, offer a promising solution to these limitations. By breaking time‐reversal symmetry, MO materials induce nonreciprocal optical responses, enabling directional control over light propagation and polarization [27]. The original spin degeneracy protected by time‐reversal symmetry is a foundational principle in topological physics, and breaking this symmetry gives rise to novel physical phenomena [28]. Integrating MO materials with BICs enables independent modulation of chirality and topological charge, offering new potential for directionally asymmetric photonic systems. The exploration of the MO materials has shown us an inspiring result about the application. The work reports a high *Q* factor > 18,300 and a giant Faraday rotation 41° under an experimentally achievable magnetic field of 2500 Oe. This demonstrates that YIG‐family materials can indeed support high‐Q resonances with strong magneto‐optical response in compact nanostructures [29].

In this letter, we demonstrate magnetically decoupled Janus BICs by a bi‐layer PhC composed of a $C_{3v}$ slab and an MO $C_{6v}$ slab, as shown in Figure 1. Our system realizes independent modulation of topological charge and chirality, which was not achieved in previous research studies. When an external magnetic field is applied as a perturbation to break time‐reversal symmetry, the degenerate BICs in the MO slab split into controllable opposite chiral modes, while retaining the robustness of asymmetric topological charges. The modified nonreciprocal TCMT with perturbation is proposed to describe the system and explain the results, including nonreciprocal polarization conversion and circular dichroism (CD). Different from the existing research studies, which adjusts the C points to realize the chirality bonding with the topological charge [24], we achieve a new path for chirality through breaking the time‐reversal symmetry.

**FIGURE 1:**
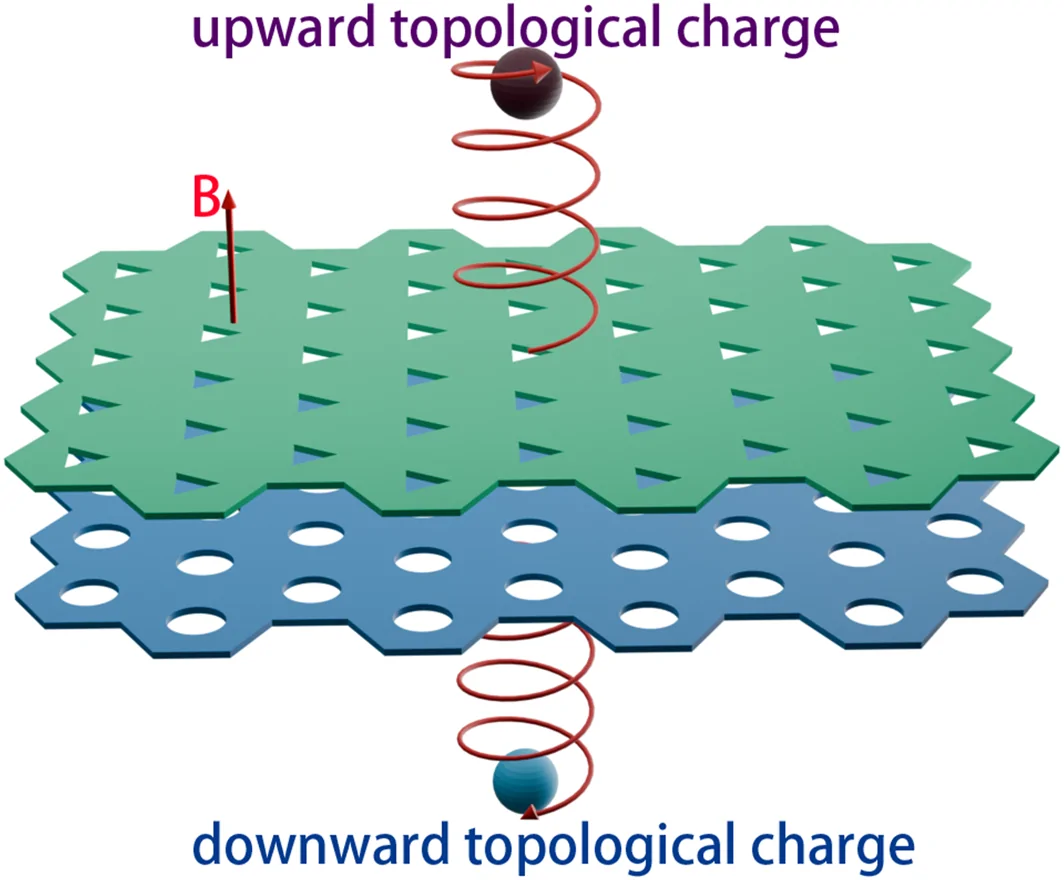
Schematic of the bi‐layer PhCs slab. With the breaking of $\sigma_{z}$ symmetry, the topological charge of upward radiation is different from that of downward. This difference can be controlled by the separation between the slabs. Under an external magnetic field, chirality appears on both sides, a property related to the direction of the external magnetic field. Topological charge and chirality are independent in modulation.

The system achieves dual tunability through independently controlling topological charge by adjusting slab separation and chirality by the external magnetic field (both magnitude and direction can influence the properties). The dual tunability plays significant roles: The topological charge ensures the robustness of light localization and propagation, whereas the chirality enables selective coupling with chiral quantum emitters or spin‐polarized particles. These features establish a foundation for reconfigurable topological photonic devices, such as chiral waveguides, nonreciprocal topological lasers, and spin–photon interfaces—where topological protection and chiral selectivity can be dynamically tuned. Different from vector lasing through conventional BICs [30], which lacks the additional chiral control and orbital angular momentum featured in our work, these chiral BICs carrying additional orbital angular momentum are more promising platforms in the future [31].

## 2 Theory Derivation and Simulation Method

The chirality of the BICs in the MO materials can be dynamically tuned by the external magnetic field. For the single $C_{6v}$ slab, it will support a pair of degenerate BICs. The degeneracy of the pair of BICs is protected by time‐reversal symmetry, which corresponds to the $E_{2}$ representation [32]. When an external magnetic field is applied perpendicular to the slab, time‐reversal symmetry is broken, as shown in Figure 2b. A band gap appears between the degenerate BICs. The doubly degenerate modes split into a pair of chiral modes with opposite pseudospins, characterized by two opposite circular polarizations corresponding to the $E_{2a}$ and $E_{2b}$ irreducible representations [33]. To describe the band gap and the generation of lifting the degeneracy under the external magnetic field, we use an effective Hamiltonian model (the details are provided in Supporting Information S1: Section 3)^1^:

(1) $$H=\omega_{0}-i\Gamma_{0}+\beta \left(k_{x}^{2}+k_{y}^{2}\right)+\lambda \left(k_{x}^{2}+k_{y}^{2}\right){\hat{\sigma }}_{z}+\Delta {\hat{\sigma }}_{y}.$$

**FIGURE 2:**
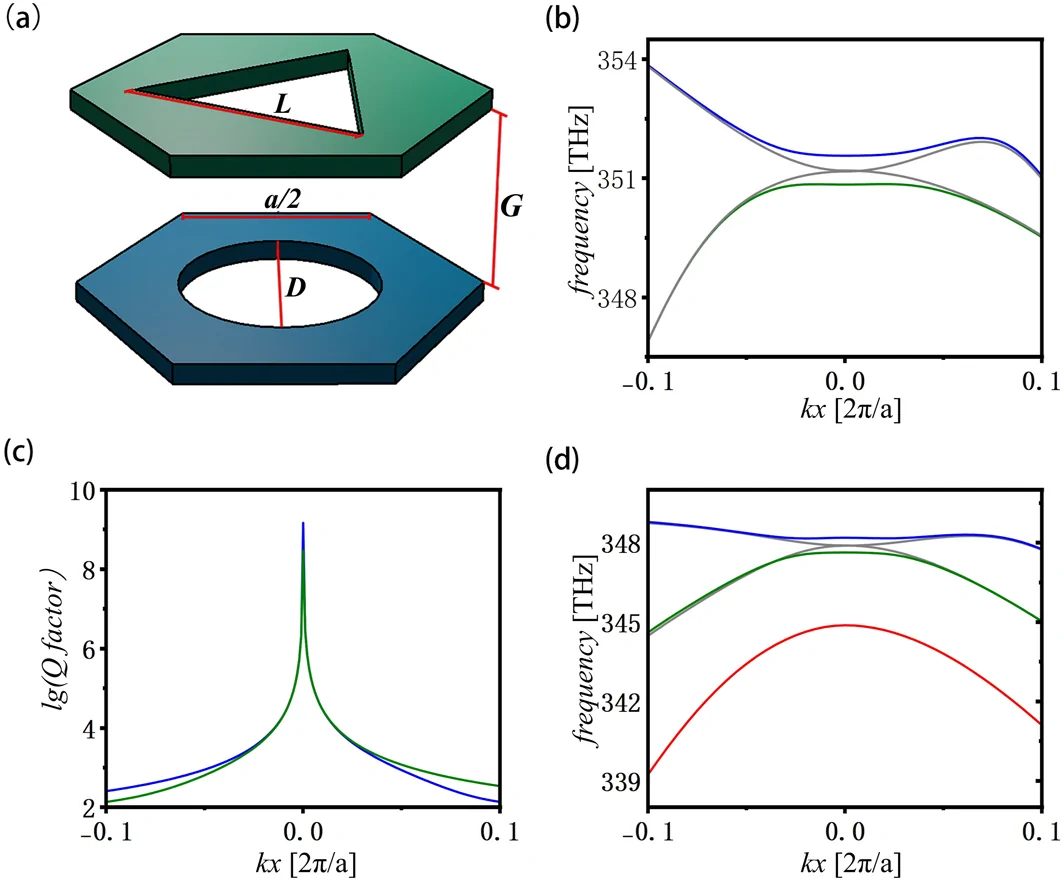
(a) Schematic of the proposed bi‐layer photonic crystal structure, composed of a nonmagnetic optical upper layer and a magnetic optical lower layer. (b) The band structures of the BICs from the simulation of a single $C_{6v}$ slab. The gray lines are the bands without an external magnetic field. The blue and green lines are the lines with an external magnetic field. (c) The Q factors of the BIC modes from the simulation. (d) The band structure of the bi‐layer PhCs slab composed of $C_{3v}$ and $C_{6v}$ slabs, which retain the chiral BICs of the $C_{6v}$ slab. The gray lines are the bands without an external magnetic field, and the blue and green lines are the bands with an external magnetic field. The red line is the band from the non‐MO materials PhCs.

Here, $\omega_{0}$ is the mean mode energy, β and λ are two constants which influence the dispersion of bands, $\Gamma_{0}$ is the radiation, and Δ represents the coupling strength, which is decided by the magnitude of the magnetic field. It can be found that the Hamiltonian is not diagonalized, and the degeneracy at the Γ point is lifted. The effective Hamiltonian exhibits the origin of the degeneracy induced by the magneto‐optical effect. The band structures of the bi‐layer PhCs composed of the $C_{3v}$ slab and the $C_{6v}$ slab are exhibited in Figure 2d.

The system consists of two slabs with $C_{6v}$ and $C_{3v}$ symmetry, respectively. When they are stacked vertically with a separation G, the $\sigma_{z}$ symmetry is broken, and the interaction between similar bands will cause C points to appear. C points are the points with specific Stokes parameters ($S_{1}=S_{2}=0,S_{3}=\pm 1$ [34]). The movements of the C points in the upward and downward directions are different, which will induce the Janus behavior [24]. Although the design can control the chirality of the system through the C points, the chirality and the topological charge are bound together. To address the challenge, we induce the MO materials in $C_{6v}$ slab to achieve a decoupled degree of chirality.

We construct the bi‐layer PhCs with the $C_{6v}$ slab and the $C_{3v}$ slab. The dielectric constant is set to $\epsilon =2.02^{2}$ for the upper slab, which is not made of MO materials. The lower slab is set as the MO materials with a diagonal relative permittivity tensor [35, 36]:

(2) $$\epsilon \left(r\right)=\left(\begin{array}{ccc} \epsilon & -i\delta & 0\\ i\delta & \epsilon & 0\\ 0 & 0 & \epsilon \end{array}\right).$$

The parameter δ (related to magneto‐optical activity) is dynamically tunable through the magnetic field, while the relative permeability is $\mu_{r}=1$. We set the lattice constant of the hexagonal lattice as a=800 (nm). The upper slab has $C_{3v}$ symmetry and contains a triangular structure at its center, with the side length of the triangle being L=0.9a. The separation between the two slabs is G=350 (nm). To form the bands with similar structures and a stable gap, which is the requirement of the Janus BICs, the thickness of the slabs should be chosen carefully. The thickness of the upper slab is $t_{1}=108$ (nm), whereas that of the lower slab is $t_{2}=100$ (nm). The diameter of the central hole in the lower slab is D=0.7a. The boundary conditions in the simulation are periodic boundary conditions.

## 3 Results and Discussion

In previous studies, intrinsic chirality has been typically achieved by tuning C points to the Γ point through structural changes [37]. By using PhCs made of magneto‐optical materials with $C_{6v}$ symmetry, we can simplify this process through the modification of an external magnetic field. Crucially, the magnetic modulation does not compromise the high‐Q factors of the BICs. The magnetic field primarily influences the $S_{3}$ component of the Stokes parameters. In other words, chirality and topological charge are decoupled. This unique feature opens up various possibilities for applications. We create a modulation platform with higher degrees of freedom. Polarization vortices, which are generated in momentum space through spin–orbit interactions, offer promising applications in vortex beam generation. Manipulation of the phase singularities in optical vortices is a critical challenge in optical communications, quantum information processing, and related fields. Notably, the periodic structures in real space lack an optical axis, which means that the periodic structures (such as Photonic crystals in the article) do not possess a globally defined anisotropic direction in real space. Consequently, polarization vortices induce a Pancharatnam–Berry phase through polarization conversion, which evolves as the polarization direction changes across momentum space [38, 39]. To address the challenge of the manipulation of the phase singularities in optical vortices, we investigate the polarization properties of our slab system at different incidence directions from downward and upward incidence. Because the system, which is a bi‐layer structure, lacks $\sigma_{z}$ symmetry, the transmission characteristics for the two propagation directions are nonreciprocal. From the analysis of the separation G in the topological charge of stacking slabs, the conclusion is that the circularly polarized points are getting closer to the Γ point, whereas the effects between the upward and downward differ. It can be speculated that the upward and the downward radiation will not merge near the Γ point simultaneously. Therefore, we can achieve different topological charges around the Γ point in opposite directions to create the manipulation of the vortices in the upward and downward directions, which is described as the Janus behavior. We chose the G as G=350nm because the C points do not merge completely but obviously differ between upward and downward for a better view to understand the evolution as shown in Figure 3. The distinct evolution pathways of the C points for upward and downward radiation in Figure 3 demonstrate the Janus behavior of our system.

**FIGURE 3:**
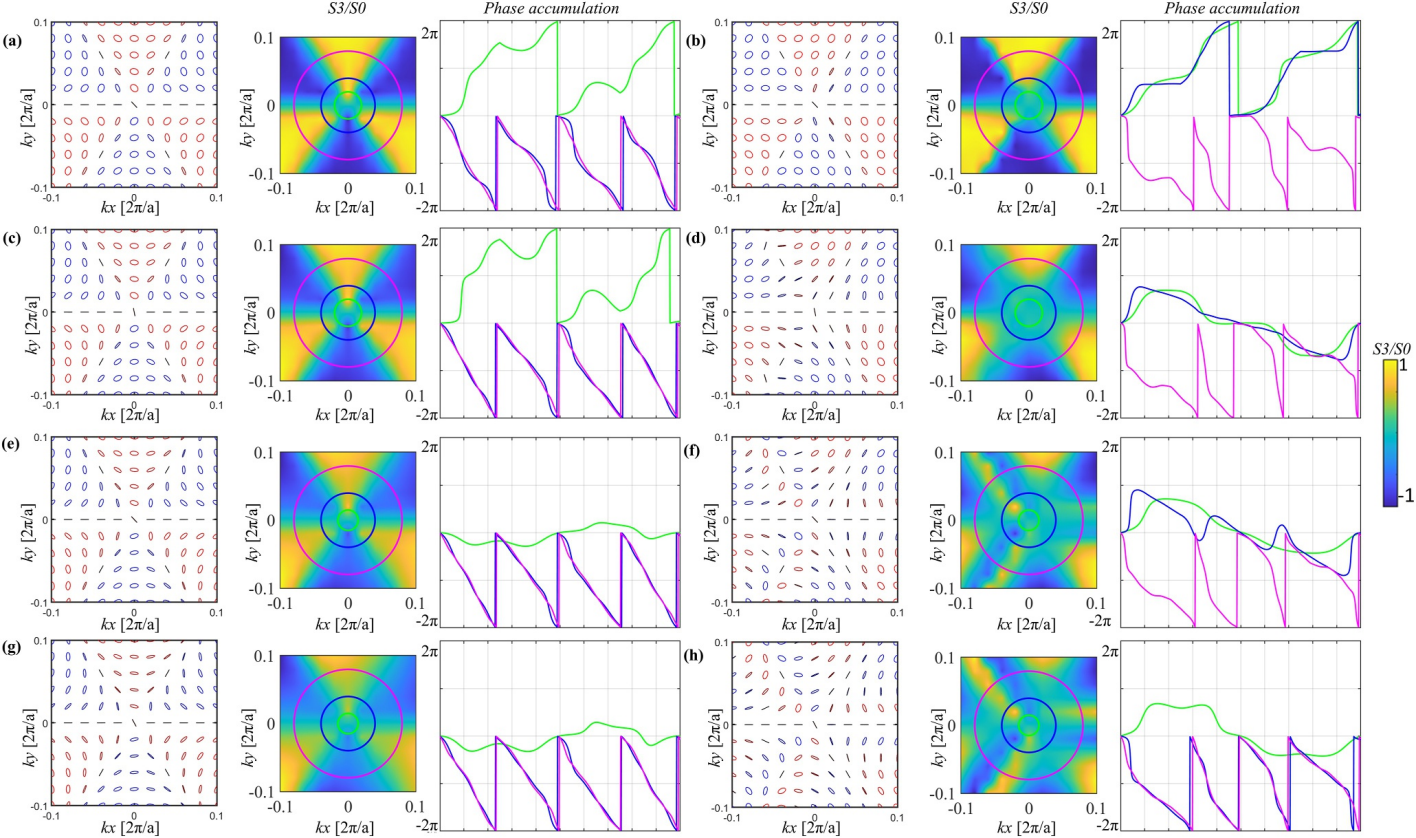
The evolution of topological charges in upward polarization and downward polarization with different values of G, respectively. The polarization ellipse, normalized $S_{3}$ parameters, and corresponding phase accumulation with different radii around the Γ point are provided. (a) The upward polarization with G=340 (nm). (b) The downward polarization with G=340 (nm). (c) The upward polarization with G=350 (nm). (d) The downward polarization with G=350 (nm). (e) The upward polarization with G=360 (nm). (f) The downward polarization with G=360 (nm). (g) The upward polarization with G=370 (nm). (h) The downward polarization with G=370 (nm).

### 3.1 Janus Behavior

BICs are recognized as topological defects in momentum space, manifesting as polarization vortices that carry topological charges. The topological charge is defined to describe the winding number of the polarization axis of leaky modes around BICs. The topological charge is defined as follows [40]:

(3) $$q=\frac{1}{2\pi }\oint_{L}dk_{\|}\cdot \nabla_{k_{\|}}\phi \left(k_{\|}\right).$$

The L is a closed loop around the BICs, $\phi \left(k_{\|}\right)$ is the azimuthal angle of the major axis of polarization state. In the condition of the Stokes parameters, the phase can be calculated as follows [41]:

(4) $$\phi \left(k_{\|}\right)=\frac{1}{2}arg\left[S_{1}\left(k_{\|}\right)+iS_{2}\left(k_{\|}\right)\right].$$

Here, $S_{i}\left(i=1,2,3\right)$ are the Stokes parameters of the far‐field polarization. The cumulative ±2π phase of the Stokes parameters corresponds to $\pm \frac{1}{2}$ topological charge. The possible topological charge of the PhCs with $C_{6v}$ symmetry is −2, while the possible topological charge of the PhCs with $C_{3v}$ symmetry is 1. More details about the symmetry and its corresponding topological charges can be found in Supporting Information S1: Section 10 (see Endnote 1). When we modulate the value of G to achieve a suitable interaction between the two slabs, their far‐field polarizations will influence each other. Focusing on the lower band of the $C_{6v}$, the difference between the topological charge induces three pairs of C points. Every C point carries the topological charge of $-\frac{1}{2}$ [16].

The evolutions of the topological charges in the different directions are provided in Figure 3. The left panels in Figure 3 correspond to the upward polarization, and the right panels correspond to the downward polarization. The values of G increase from 340(nm) to 370(nm) by the steps of 10(nm). When the values of G=340(nm), the topological charge on both sides becomes almost similar. In this situation, the interaction between the two slabs becomes so strong that the symmetry should be considered one $C_{3v}$ cavity instead of combinations of different symmetries. When the values of G=370(nm), the interaction between the slabs is weak, causing the topological charge on both sides to also be similar to that of a single $C_{6v}$ slab. Therefore, we choose G=350(nm) because the topological charges on different sides are distinct.

Because of the topological charge difference between the $C_{3v}$ slab and the $C_{6v}$ slab, the C points emerge. Their actions are distinct in the upward and downward movements to form the Janus behavior. Figure 4 provides the specific movements of the C points. When two slabs get closer, the C points tend to merge together at the Γ point. The C points move faster in the downward direction with the increase of the G. As a result, the same region in different directions will carry distinct topological charges.

**FIGURE 4:**
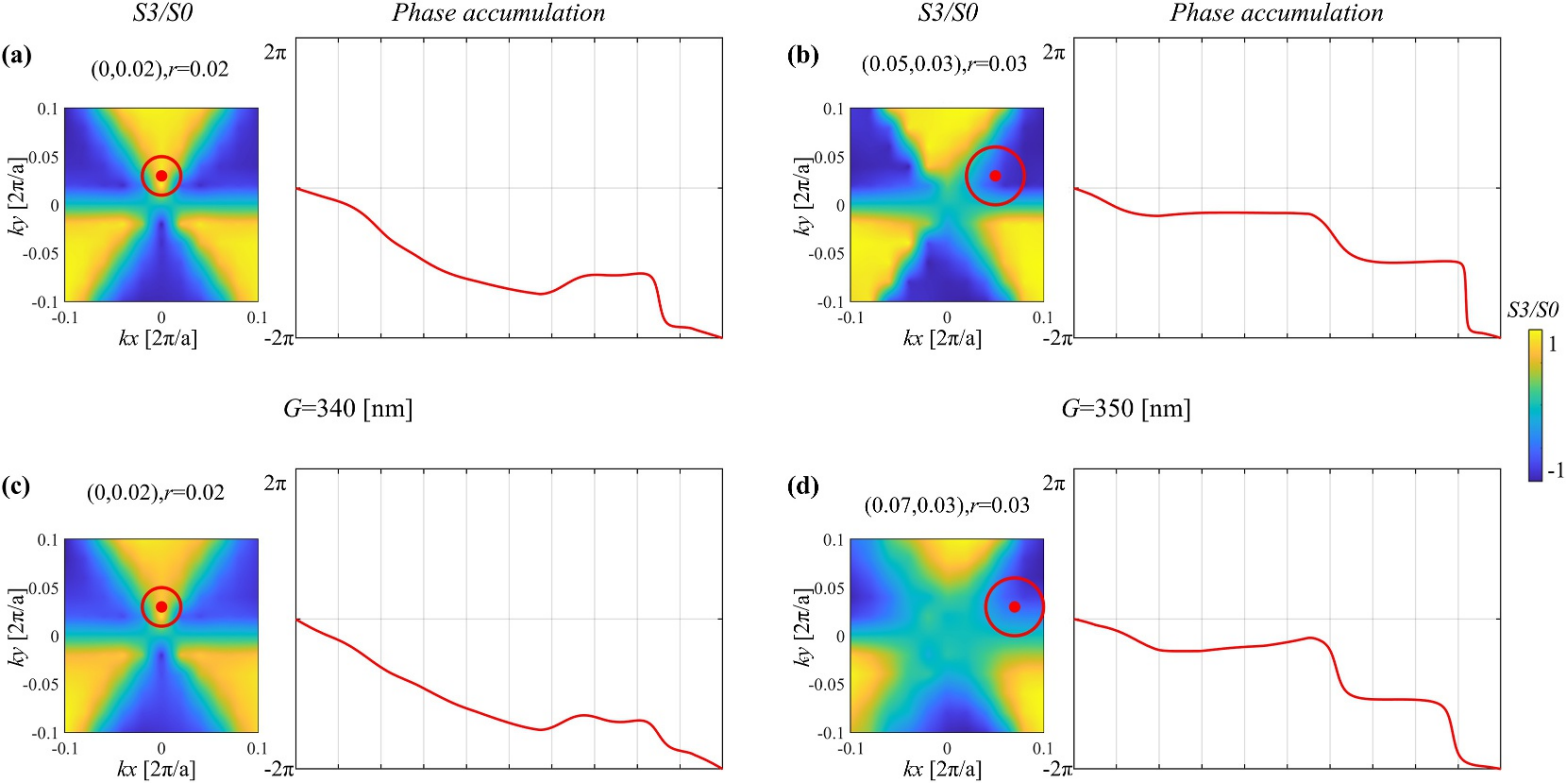
The diverse movements of the C points in the upward and downward directions. The corresponding phase accumulation along the circle could prove the C points with −1/2 topological charge. Panels (a) and (b) exhibit the C points in the upward, and panels (c) and (d) exhibit the downward. The influences of G are not the same in upward and downward, so that the C points could be controlled to form different topological charges.

### 3.2 Independent Modulation Through the External Magnetic Field

Such a stacking of the two slabs is a breaking of symmetry in $\sigma_{z}$. The intriguing results come when we apply an external magnetic field to the PhCs, as in Figure 5. We explore the evolution of the far‐field radiation of polarization distribution in the momentum space under the different orientations of the external magnetic field. Figure 5a,b is the case where the magnetic fields are applied along the +z direction (corresponding to a positive δ). Comparing them to the cases without an external magnetic field, which are shown as Figure 3c,d, the external magnetic field breaks the time‐reversal symmetry and introduces an additional chirality. The external magnetic field selects 3 C points carrying $-\frac{1}{2}$ topological charge in the upward direction; these C points merge with the basic 1 charge in the Γ point to form a region with chirality. Switching the direction of the external magnetic field could provide the fixed topological charge with different chirality. In the downward direction, 3 C points with opposite chirality are absent from the region, so that the topological charge in the whole *k*‐space becomes $-\frac{1}{2}$ with chirality.

**FIGURE 5:**
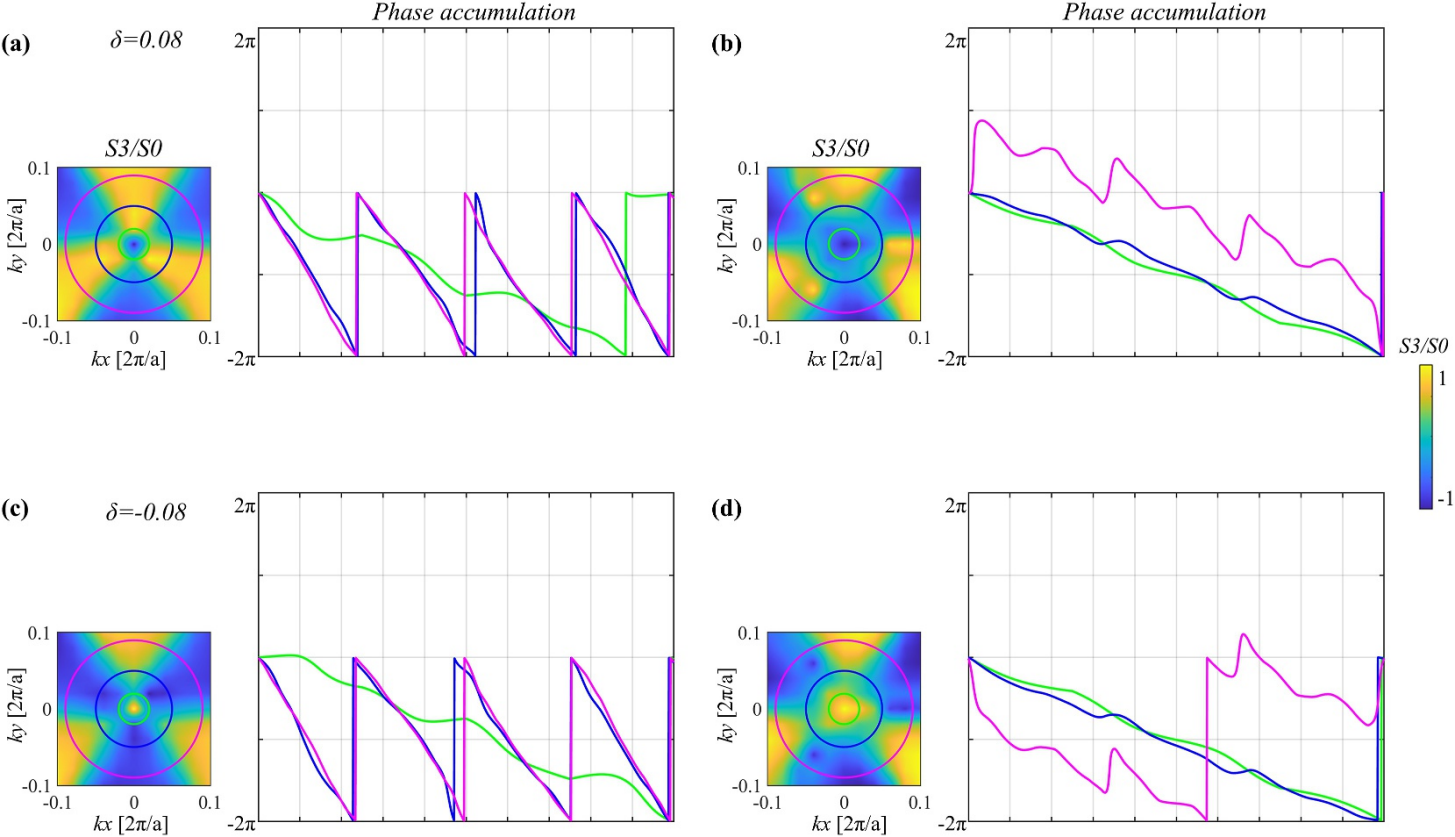
The example of decoupled modulation of topological charge. G=350(nm), the topological charge is fixed and the direction of the external magnetic field only controls the chirality. Panels (a) and (b) are the upward and downward polarization with δ=0.08. Panels (c) and (d) are the upward and downward polarization with δ=−0.08.

Intriguingly, the topological charges of the BICs remain robust even as the external magnetic field varies.

In Figure 6, we provide the upward polarization under a series of values of the magnetic field. Under the changes of the external magnetic field, the total topological charge remains −2, which is the topological charge the $C_{6v}$ slab carries. That is the behavior governed by the conservation law of topological charges in photonic systems. Figure 6 demonstrates that the magnetic field mainly modifies the chirality while leaving the topological charge unchanged, thereby supporting the decoupled‐control claim.

**FIGURE 6:**
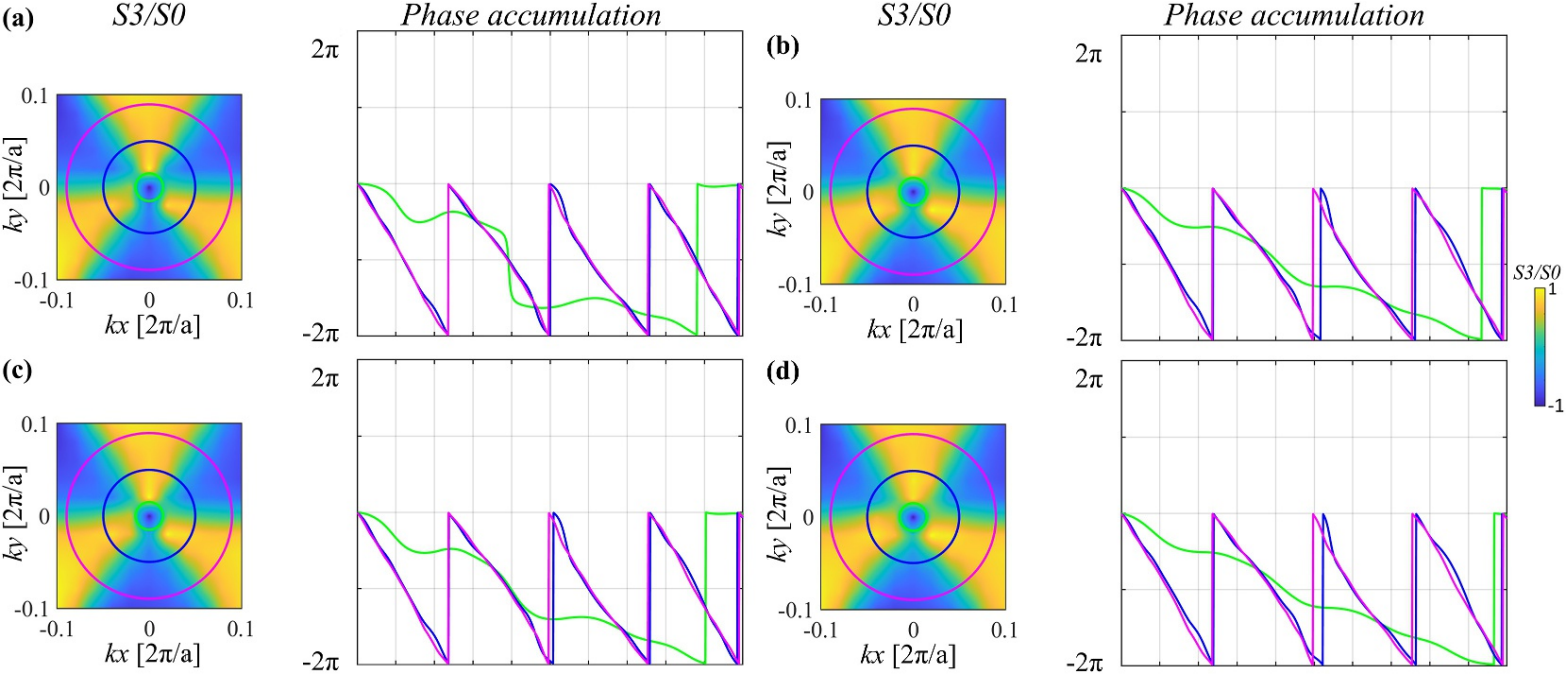
The robustness of topological charge under the perturbation of the δ. The normalized $S_{3}$ parameters and corresponding phase accumulation with different radii around the Γ point are provided. (a) δ=0.02. (b) δ=0.04.(c) δ=0.06. (d) δ=0.08.

Achieving reliable light–matter interactions in topological photonic systems needs the robustness of topological charge. As the momentum approaches the central Γ point in momentum space, the polarization state evolves into a specific circular polarization, and the winding of the azimuthal angle transforms into a winding phase. In this regime, the different topological charge corresponds to opposite chirality. Because the fixed G provides a Janus topological charge on different sides, the direction of the external magnetic field will introduce a chirality independent of the manipulation of the Janus topological charge. The system exhibits independent paths in controlling chirality and topological charge. These results indicate that the specific spin matches an orbital angular momentum of light, enabling deterministic control over the polarization and propagation of photons.

### 3.3 Optical Properties

We derived the TCMT of the MO materials with typical nonreciprocity properties. We present the polarization conversion and CD from both sides of the slab to demonstrate the nonreciprocal behavior of the Janus BICs with tunable chirality. Because the infinite *Q* factor of the BICs cannot be observed on the spectrum, we need q‐BICs instead of BICs. All results correspond to an oblique incidence angle of 2°. The CD corresponds to the different absorption of the LCP and RCP. The efficiencies of polarization conversion are also shown for the LCP and RCP, respectively. Because the oblique incidence may influence the $C_{2}^{2}$ symmetry, which is significant in the derivation of the TCMT, we discuss the reliability of the approximation under near‐normal incidence in Supporting Information S1: Section 7 (see Endnote 1). Figure 7a,b shows the polarization under the forward incidence. We derive the polarization conversion efficiency as follows (the derivation of TCMT and its conclusion of properties can be found in Supporting Information S1: Sections 5 and 8 [see Endnote 1]):

(5) $$\zeta_{f,L\to R}=|J_{RL}^{T,f}{|}^{2}=|\frac{d_{R}^{u}k_{L}^{d}}{\eta }{|}^{2},$$

(6) $$\zeta_{f,R\to L}=|J_{LR}^{T,f}{|}^{2}=|\frac{d_{L}^{u}k_{R}^{d}}{\eta }{|}^{2},$$

(7) $$\zeta_{b,L\to R}=|J_{RL}^{T,b}{|}^{2}=|\frac{d_{R}^{d}k_{L}^{u}}{\eta }{|}^{2},$$

(8) $$\zeta_{b,R\to L}=|J_{LR}^{T,b}{|}^{2}=|\frac{d_{L}^{d}k_{R}^{u}}{\eta }{|}^{2}.$$

where $\eta =i\left(\omega_{0}-\omega \right)+\gamma_{0}$, $J_{\alpha \beta }\left(\alpha ,\beta =L,R\right)$ are elements of Jones matrix. T means transmission. The words f and b mean forward and backward, respectively.

**FIGURE 7:**
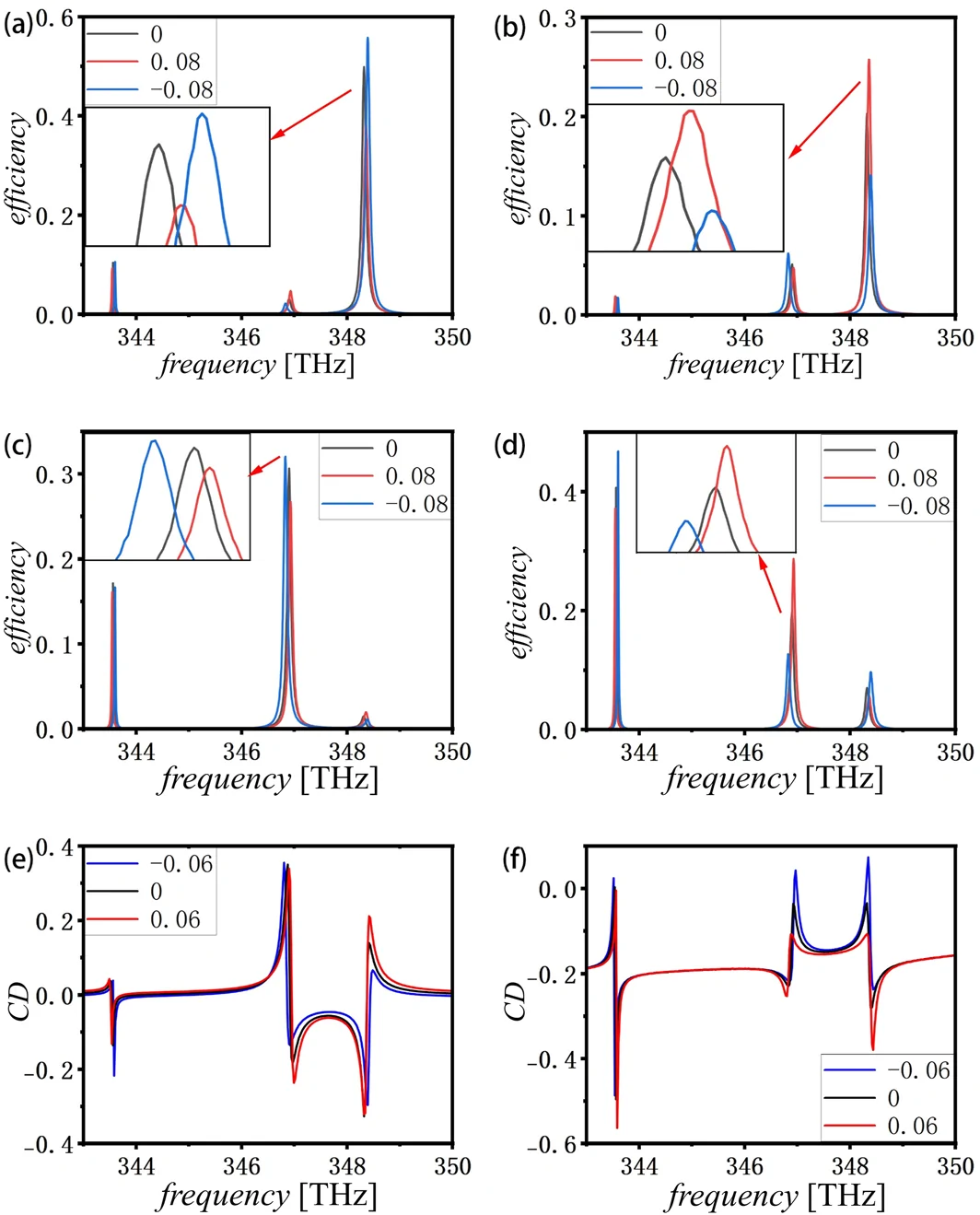
(a) Polarization conversion from LCP to RCP with forward incidence. (b) Polarization conversion from RCP to LCP with forward incidence. (c) Polarization conversion from LCP to RCP with backward incidence. (d) Polarization conversion from RCP to LCP with backward incidence. (e) CD with forward incidence. (f) CD with backward incidence. The small panels are the enlarged peaks of the polarization conversion. The enlarged version can be seen in Supporting Information S1: Section 11 (see Endnote 1).

The polarization conversion efficiency, which is strongly correlated with the resonance frequency, exhibits nonreciprocal characteristics attributable to the MO materials. When the incidence conditions change, the dominant wavelength exhibits a corresponding change. Comparing the same conversion process under different incidence conditions reveals that there are differences between the peak of backward RCP conversion and the peak of forward RCP conversion, both in terms of position and intensity. Similarly, under the magnetic fields of +z and −z directions, the RCP conversion efficiencies are enhanced and suppressed, respectively. This behavior stems from the time‐reversal symmetry breaking induced by MO materials, which modulates resonance responses under varying external conditions, affecting conversion efficiency.

The tunable nonreciprocal polarization conversion holds significant potential for practical applications in integrated photonics. Such properties can be exploited in the design of compact optical isolators, which are essential components in high‐power laser systems to prevent back‐reflected light from damaging source devices. Additionally, the field‐dependent modulation of circular polarization conversion efficiency could enable the development of magnetically controlled optical switches, where polarization state manipulation through external magnetic fields allows for dynamic routing of optical signals in communication networks.

The wavelength sensitivity under varying incidence conditions further suggests applicability in wavelength‐selective nonreciprocal devices, such as polarization‐dependent filters, which could enhance signal processing capabilities in photonic integrated circuits. CD is a significant index in optical designs. CD spectroscopy measures the difference in absorption of LCP and RCP. Its primary application is determining the secondary structure composition of structures. It is also widely used to study conformational stability, folding dynamics, and molecular interactions. The derivations from the TCMT exhibit the CD in the following form:

(9) $$CD^{f}=|t+\frac{d_{R}^{u}k_{R}^{d}}{\eta }{|}^{2}+|\frac{d_{L}^{u}k_{R}^{d}}{\eta }{|}^{2}-|t+\frac{d_{L}^{u}k_{L}^{d}}{\eta }{|}^{2}-|\frac{d_{R}^{u}k_{L}^{d}}{\eta }{|}^{2},$$

(10) $$CD^{b}=|t+\frac{d_{R}^{d}k_{R}^{u}}{\eta }{|}^{2}+|\frac{d_{L}^{d}k_{R}^{u}}{\eta }{|}^{2}-|t+\frac{d_{L}^{d}k_{L}^{u}}{\eta }{|}^{2}-|\frac{d_{R}^{d}k_{L}^{u}}{\eta }{|}^{2}.$$

where $\eta =i\left(\omega_{0}-\omega \right)+\gamma_{0}$, $J_{\alpha \beta }\left(\alpha ,\beta =L,R\right)$ are elements of Jones matrix. T means transmission. The words f and b mean forward and backward, respectively. By considering the magnitudes of the $d_{L,R}^{u,d}$, which are related to the terms of MO effect in the dielectric constants, the efficiency of polarization conversion and CD will not be higher than other works, but still are great evidence of the Janus behavior and non‐reciprocity. The modulation of magnetic fields on CD arises from the non‐reciprocal coupling coefficients K and D. As shown in Figure 7e,f, a magnetic field along the +z axis suppresses the CD intensity under forward incidence. In contrast, the CD intensity is enhanced under backward incidence. This directional dependence of CD response under a magnetic field further confirms the nonreciprocal feature induced by the breaking of the time‐reversal symmetry. Such directional modulation of CD by magnetic fields provides promising possibilities for applications in high‐sensitivity magnetic field sensing, where the differential CD response under forward or backward incidence could enable precise detection under varying magnetic field directions and magnitudes. It may facilitate the development of chiral optical switches with directional selectivity, enhancing signal routing capabilities in chiral photonics and quantum communication systems.

## 4 Conclusion

The system demonstrates the interplay among symmetry breaking of structures, magnetic field modulation of the time‐reversal symmetry, and topological physics in photonic systems. This work demonstrates independently tunable chiral bound states in the continuum realized in a bi‐layer photonic crystal structure, where a $C_{3v}$ symmetry slab and a magneto‐optical $C_{6v}$ symmetry slab enable decoupled control of topological charges through separation between the slabs and chirality through an external magnetic field. The resulting Janus BICs exhibit nonreciprocal polarization conversion and circular dichroism. Both of them are essential for chiral light–matter interactions. This platform advances reconfigurable topological photonics, with potential applications in magnetically tunable spin–photon interfaces. The decoupled control of chirality and topology may benefit chiral single‐photon sources (e.g., directional quantum emission), for which coupling to emitters remains a subject for future study [21]. This work paves the way for exploring nonreciprocal MO systems and their topological properties in both theory and device design. The work also offers advantages in nonreciprocal devices.

## Author Contributions

**Yadong Yue:** conceptualization, investigation, writing – original draft, visualization, writing – review and editing, validation, methodology, software, formal analysis, data curation, supervision. **Du Li:** methodology, software. **Kun Liang:** funding acquisition, project administration, writing – review and editing, resources. **Li Yu:** funding acquisition, project administration, writing – review and editing, resources.

## Supporting information


Supporting Information S1

## Data Availability

Data sharing is not applicable to this article as no datasets were generated or analyzed during the current study.

## Endnotes

^1^
Supporting Information S1 includes the parts of the analysis of $c_{3v}$ and $c_{6v}$ PhCs based on group theory, break of time reversal symmetry, the Hamiltonian model of the MO PhCs, nonreciprocal coupling coefficients and dynamic equation in a PhCs made of magnetic‐optical materials, nonreciprocal temporal coupled‐mode theory model, optical properties of the PhCs, far‐field polarization and Pancharatnam–Berry phase, the relationship between topological charge and symmetry and enlarged insets in Figure 7 with Refs. [33, 34, 42, 43].
