# Supplementary material for: Magnetically Decoupled Janus Bound States in the Continuum for Chirality–Topology Control
Source: Nanophotonics. 2026 May 27;15(11):e70134. doi: 10.1002/nap2.70134 (PMC13642532; doi:10.1002/nap2.70134)
Supplement: Supplementary file 1 [file NAP2-15-e70134-s001.pdf]

Supplemental Material for Magnetically Decoupled Janus Bound States in the Continuum for Chirality-Topology Control

April 9, 2026

1 The analysis of C_{3v} and C_{6v} PhCs based on Group Theory

The E_2 representation of a C_6 photonic crystal slab with time-reversal symmetry is a two-dimensional representation. The E_2 representation is composed of two one-dimensional irreducible representations E_{2a} and E_{2b}, whose degeneracy is ensured by time-reversal symmetry.

When the time-reversal symmetry is broken by an external magnetic field, the degenerate modes split into a pair of single states, which correspond to E_{2a} and E_{2b}. Here we get a character table of E_2 representation[1].

Representations		E	C_6	C_3	C_2	$(C_3)^2$	$(C_6)^5$
E_2	E_{2a}	1	ω^2	ω^4	1	ω^2	ω^4
	E_{2b}	1	ω^4	ω^2	1	ω^4	ω^2

Table 1: Character table of E_2 representation .$\omega = e^{2\pi i/6}$

From the table, we could obtain the winding phase profiles of the modes.For E_{2a},when rotate around the z axis for $\pi/3$, the mode will be :

$$R(\pi/3)\psi(\mathrm{r}) = w^2\psi(\mathbf{r}) = \psi(R^{-1}\mathbf{r}). \tag{S1}$$

Therefore, the phase will increase by $\arg(\omega^2) = 2\pi/3$ under the C_6 symmetry. When rotating the whole 2π, the phase will wind by -4π. It is obvious as a contrast that the phase will wind 4π under E_{2b} around the z axis.

2 Break Of Time Reversal Symmetry

For the electromagnetic waves in photonic crystals, the equation of the wave is :

$$[\nabla \times (\epsilon(\mathbf{r})^{-1}\nabla \times) + \frac{1}{c^2}\frac{\partial^2}{\partial t^2}]\mathbf{H}(\mathbf{r},t) = 0. \tag{S2}$$

When remain the time-reversal symmetry, the ϵ satisfy $\epsilon(\mathbf{r}) = \epsilon(\mathbf{r})^*$. But for MO materials, the dielectric constants satisfy[2]:

$$\epsilon(\mathbf{r}) = \begin{pmatrix} \epsilon & -i\delta & 0 \\ i\delta & \epsilon & 0 \\ 0 & 0 & \epsilon \end{pmatrix}. \tag{S3}$$

Since $\epsilon(\mathbf{r}) \neq \epsilon(\mathbf{r})^*$, the time-reversal symmetry is broken[3].

3 The Hamiltonian model of the MO PhCs

Without an external magnetic field, the modes of $E-2$ representation can be described as follows: an effective Hamiltonian model:

$$\mathbf{H}_{eff} = \omega_0 - i\mathbf{\Gamma}_0(\mathbf{k}) + \beta(k_x^2 + k_y^2) + \lambda(k_x^2 + k_y^2)\hat{\sigma_z}. \tag{S4}$$

Here ω_0 is the mean mode energy,$\beta$and λ are two constants which influence the dispersion of bands. $\mathbf{\Gamma}_0$ is the radiation loss which depends on the wave vector $\mathbf{k} = (k_x, k_y)$, which is related to the coupling coefficients $\mathbf{D}_0$ from the coupled mode theory :

$$2(\mathbf{\Gamma}_0) = \mathbf{D}_0^\dagger \mathbf{D}. \tag{S5}$$

With the external magnetic field, the time reversal symmetry is broken, and it acts like a perturbation term in the Hamiltonian :

$$\mathbf{H_{eff}} = \omega_0 - i\mathbf{\Gamma}_0(\mathbf{k}) + \beta(k_x^2 + k_y^2) + \lambda(k_x^2 + k_y^2)\hat{\sigma_z} + \Delta\hat{\sigma_y}. \tag{S6}$$

Here Δ represents the coupling strength, which is decided by the magnitude of the magnetic field (value of the δ in dielectric constants). Since the Hamiltonian is not diagonalized, the degeneracy at the Γ point vanished. The derivation of the effective Hamiltonian exhibits the origin of the degeneracy induced by the magneto-optical effect.

4 Non-reciprocal coupling coefficients and dynamic equation in a PhCs made of magnetic-optical materials

First, we consider a common situation where we use orthogonal modes to expand the electric field:

$$\langle \vec{E_n} | \, | \vec{E_m} \rangle > \int_v \vec{E_m}^* \epsilon \vec{E_n} dV = 2U_n \delta_{mn}. \tag{S7}$$

Here U_n represents the energy of the mode, which is:

$$U_n = \frac{1}{2}\int_v \epsilon |\vec{E_n}|^2 dV. \tag{S8}$$

After normalization:

$$\langle \vec{E_m} | \, | \vec{E_n} \rangle >= \delta_{mn}. \tag{S9}$$

Then we expand the electric field by these bases:

$$|\vec{E}(t)\rangle = \Sigma_n [a_n(t) \, |\vec{E_n}(t)\rangle \, e^{-i\omega_n t}] + const. \tag{S10}$$

From Maxwell's equations without source, there is:

$$\nabla \times \nabla \times |\vec{E}(t)\rangle + \mu_0 \epsilon_t \frac{\partial^2}{\partial t^2} |\vec{E}(t)\rangle = 0. \tag{S11}$$

Where:

$$\epsilon_t = \epsilon + \Delta\epsilon$$

The first item takes the form of a diagonal, and the second item takes the form

$$\begin{pmatrix} 0 & -i\delta \\ i\delta & 0 \end{pmatrix}. \tag{S12}$$

which means the external magnetic field. Then the equation becomes:

$$\nabla \times \nabla \times |\vec{E}(t)\rangle + \mu_0 \epsilon \frac{\partial^2}{\partial t^2} |\vec{E}(t)\rangle = -\mu_0 \frac{\partial^2}{\partial t^2} \Delta\epsilon \, |\vec{E}(t)\rangle \, . \tag{S13}$$

Here, because of the slowly varying envelope approximation, which means:

$$|\dot{a_n}| \ll \omega_n |a_n|, |\ddot{a_n}| \ll \omega_n |\dot{a_n}|. \tag{S14}$$

After simplification, the equation above becomes:

$$-2i\epsilon \Sigma_n \omega_n \dot{a_n}(t) \, |\vec{E_n}\rangle \, e^{-i\omega_n t} = \Sigma_n \omega_n^2 \Delta\epsilon a_n(t) \, |\vec{E_n}\rangle \, e^{-i\omega_n t}. \tag{S15}$$

Now we multiply the $\langle \vec{E_m}| \, e^{i\omega_m t}$ on the left of both sides, as the orthogonality of the $\langle \vec{E_m}|$and $|\vec{E_n}\rangle$, there is:

$$-4i\omega_m \dot{a_m}(t) = \Sigma_n \omega_n^2 \, \langle \vec{E_m}| \, \Delta\epsilon \, |\vec{E_n}\rangle \, a_n(t) e^{i(\omega_m - \omega_n)t}. \tag{S16}$$

Noted that $\omega_m \cong \omega_n$,and here we definite the item:

$$\kappa_{mn} = \frac{\omega_m}{2} \, \langle \vec{E_m}| \, \Delta\epsilon \, |\vec{E_n}\rangle \, . \tag{S17}$$

Combining the effect of coupling and resonance frequency ω_m and corresponding decay rates γ_m, here is the dynamic equation of a non-degenerate resonance in a PhCs made of MO materials:

$$\dot{a_m}(t) = (-i\omega_m - \gamma_m) a_m(t) + i\Sigma_n \kappa_{mn} a_n(t) e^{i(\omega_m - \omega_n)t}. \tag{S18}$$

5 Non-reciprocal Temporal coupled-mode theory model

Here, we derive the scattering matrix from the dynamic equations. For a straightforward derivation, we write the equation as[4]:

$$\dot{A} = (-i\omega_0 - \gamma_0)A + K^T a. \tag{S19}$$

In this equation, A is the normalized resonance amplitude, ω_0 is the resonance frequency, and γ_0 is the corresponding decay rate. The column vectors $a = (a_s^u, a_s^d, a_p^u, a_p^d)^T$is the amplitude of the incident plane wave while $b = (b_s^u, b_s^d, b_p^u, b_p^d)^T$ is the amplitude of the outgoing plane wave. The subscripts denote the polarization as s-polarization which matches $\hat{s} = \hat{z} \times \hat{k}$, and polarization matches $\hat{p} = \hat{s} \times \hat{k}$, where $\hat{k}$ is the upward-coming direction of plane waves. The coupling matrix $K = (k_s^u, k_s^d, k_p^u, k_p^d)^T$ is composed of coupling coefficients from the incident wave to the resonance mode, and the coupling matrix $D = (d_s^u, d_s^d, d_p^u, d_p^d)^T$is composed of coupling coefficients from the resonance mode to the outgoing wave. The scattering matrix S is defined as $S = C + \frac{DK^T}{\eta}$, where $\eta = i(\omega_0 - \omega) + \gamma_0$ to describe the full scattering of the PhCs. Notably, C can be approximated by the scattering of a homogeneous slab. The system should obey some rules. The first one is energy conservation, which means that in the absence of the incident wave and material loss, the energy dissipation of the resonance should be taken by the outgoing wave. This can be written as $d(A^\dagger A)/dt = -b^\dagger b$, inducing the coupling coefficients related to the decay rate through the energy conservation:

$$D^\dagger D = 2\gamma_0. \tag{S20}$$

Then we noticed that the system retains the C_2^z rotation symmetry, so we still satisfy through angular momentum conservation:

$$CD^* = -D. \tag{S21}$$

Because of the MO materials which break the time-reversal symmetry and the characteristics of the ϵ, compared to the definition of K and D, there comes:

$$K = D^*. \tag{S22}$$

Because there is no coupling between s-polarization and p-polarization, on this basis, we could write the matrix C as:

$$C = \begin{pmatrix} C_s & 0 \\ 0 & C_p \end{pmatrix} = \begin{pmatrix} r_s & t_s & 0 & 0 \\ t_s & r_s & 0 & 0 \\ 0 & 0 & r_p & t_p \\ 0 & 0 & t_p & r_p \end{pmatrix}. \tag{S23}$$

6 Approximation of near-normal incidence

For near-normal incidence, there is $r_s = r_p = r, t_s = t_p = t$ Then follow the definition of S:

$$S = C + \frac{DK^T}{\eta} = \begin{pmatrix} R^b & T^f \\ T^b & R^f \end{pmatrix}. \tag{S24}$$

Therefore, the reflection and transmission matrices under forward propagating incidence are:

$$R^f = \begin{pmatrix} r_s + \frac{d_s^d k_s^d}{\eta} & \frac{d_s^d k_p^d}{\eta} \\ \frac{d_p^d k_d^d}{\eta} & r_p + \frac{d_p^d k_p^d}{\eta} \end{pmatrix}. \tag{S25}$$

and

$$T^f = \begin{pmatrix} t_s + \frac{d_s^u k_s^d}{\eta} & \frac{d_s^u k_p^d}{\eta} \\ \frac{d_p^u k_s^d}{\eta} & t_p + \frac{d_p^u k_p^d}{\eta} \end{pmatrix}. \tag{S26}$$

As we discuss the effect of the left-handed and right-handed circularly polarized light, we adopt Jones vectors to describe the polarization.In the discussion above, s-polarization is $|s\rangle = (0\ 1)^T$ and p-lorization is $|p\rangle = (1\ 0)^T$. Then the left-handed circularly polarized (LCP) state is $|L\rangle = \frac{|p\rangle + i|s\rangle}{\sqrt{2}}$ and the right-handed circularly polarized (RCP) state is $|R\rangle = \frac{|p\rangle - i|s\rangle}{\sqrt{2}}$. The use of the unitary transformation in the form of:

$$U = \frac{1}{\sqrt{2}} \begin{pmatrix} 1 & i \\ 1 & -i \end{pmatrix}. \tag{S27}$$

We can calculate the Jones matrices by the transformation U.To make it intuitive, we calculated the coupling matrix under the basis of $|L\rangle$ and $|R\rangle$:

$$d_L^\beta = \frac{1}{\sqrt{2}}(d_p^\beta + i d_s^\beta), \tag{S28}$$

$$d_R^\beta = \frac{1}{\sqrt{2}}(d_p^\beta - i d_s^\beta), \tag{S29}$$

$$k_L^\beta = \frac{1}{\sqrt{2}}(k_p^\beta + i k_s^\beta), \tag{S30}$$

$$k_R^\beta = \frac{1}{\sqrt{2}}(k_p^\beta - i k_s^\beta). \tag{S31}$$

Here $\beta = u, d$ represents a different side. Then the Jones matrix of the reflection can be read as:

$$J_R^f = \begin{pmatrix} J_{LL}^{R,f} & J_{LR}^{R,f} \\ J_{RL}^{R,f} & J_{RR}^{R,f} \end{pmatrix}. \tag{S32}$$

The elements of the matrix are:

$$J_{LL}^{R,f} = r + \frac{d_L^d k_L^d}{\eta}, \tag{S33}$$

$$J_{LR}^{R,f} = \frac{d_L^d k_R^d}{\eta}, \tag{S34}$$

$$J_{RL}^{R,f} = \frac{d_R^d k_L^d}{\eta}, \tag{S35}$$

$$J_{RR}^{R,f} = r + \frac{d_R^d k_R^d}{\eta}. \tag{S36}$$

And the Jones matrix of the transmission is

$$J_T^f = \begin{pmatrix} J_{LL}^{T,f} & J_{LR}^{T,f} \\ J_{RL}^{T,f} & J_{RR}^{T,f} \end{pmatrix}. \tag{S37}$$

The elements of the matrix are:

$$J_{LL}^{T,f} = t + \frac{d_L^u k_L^d}{\eta}, \tag{S38}$$

$$J_{LR}^{T,f} = \frac{d_L^u k_R^d}{\eta}, \tag{S39}$$

$$J_{RL}^{T,f} = \frac{d_R^d k_L^d}{\eta}, \tag{S40}$$

$$J_{RR}^{T,f} = t + \frac{d_R^u k_R^d}{\eta}. \tag{S41}$$

It is obvious that the Jones matrix is non-reciprocal. Here, we discuss the reflection of the different incidences of the light to show this conclusion: Similarly, on the basis of s-p polarization, the matrices have the form of:

$$R^b = \begin{pmatrix} r_s + \frac{d_s^u k_s^u}{\eta} & \frac{d_s^u k_p^u}{\eta} \\ \frac{d_p^u k_s^u}{\eta} & r_s + \frac{d_p^u k_p^u}{\eta} \end{pmatrix}. \tag{S42}$$

and on the basis of RCP and LCP polarization we get the Jones matrix:

$$J_R^b = \begin{pmatrix} r + \frac{d_L^u k_L^u}{\eta} & \frac{d_L^u k_R^u}{\eta} \\ \frac{d_R^u k_L^u}{\eta} & r + \frac{d_R^u k_R^u}{\eta} \end{pmatrix}. \tag{S43}$$

The reciprocal request the Jones matrix satisfy:

$$J_T^b = \sigma_x (J_T^f)^T \sigma_x, \tag{S44}$$

$$\sigma_x = \begin{pmatrix} 0 & 1 \\ 1 & 0 \end{pmatrix}. \tag{S45}$$

Obviously, in this situation, the Jones matrix shows the Non-reciprocity.

7 The rationality of the approximation

Since we derived the previous equation under normal incidence and approximated the 2° of incidence as near-normal incidence, here we estimate whether this approximation is reasonable. Given the parameters of frequency $f = 350\,[\mathrm{THz}]$ and lattice constant $a = 840\,[\mathrm{nm}]$, calculations for an incident angle of 2° yield: the transverse wavevector $k_x = \frac{2\pi}{\lambda}\sin 2^\circ \approx 2.56 \times 10^5\,[\mathrm{rad/m}]$ occupies about 6.85% of the Brillouin zone boundary π/a; the relative shift in resonance frequency is $\Delta f/f \approx \frac{1}{2}\beta(k)\left(\frac{k_x a}{2\pi}\right)^2$; and the relative change in coupling coefficients is $\Delta d/d \sim \sin 2^\circ$. All of these changes are bearable in the approximation. These minor perturbations indicate that symmetry breaking is weak and the frequency response remains largely preserved. Therefore, treating 2° oblique incidence as near-normal incidence is reasonable.

8 Optical properties of the PhCs

It is notable that the PhCs have some unique properties regarding transmission and polarization. Now we discuss the circular dichroism and polarization conversion in this situation. From the Jones matrix, the transmittance can be written as:

$$T_{LCP} = |t + \frac{d_L^u k_L^d}{\eta}|^2 + |\frac{d_R^u k_L^d}{\eta}|^2, \tag{S46}$$

$$T_{RCP} = |t + \frac{d_R^u k_R^d}{\eta}|^2 + |\frac{d_L^u k_R^d}{\eta}|^2. \tag{S47}$$

Since then the CD will be calculated:

$$CD^{forward} = T_{RCP} - T_{LCP} = |t + \frac{d_R^u k_R^d}{\eta}|^2 + |\frac{d_L^u k_R^d}{\eta}|^2 - |t + \frac{d_L^u k_L^d}{\eta}|^2 - |\frac{d_R^u k_L^d}{\eta}|^2. \tag{S48}$$

For the downward situation there is:

$$CD^{backward} = |t + \frac{d_R^d k_R^u}{\eta}|^2 + |\frac{d_L^d k_R^u}{\eta}|^2 - |t + \frac{d_L^d k_L^u}{\eta}|^2 - |\frac{d_R^d k_L^u}{\eta}|^2. \tag{S49}$$

Noticed that the CD of backward is different from the one of upward. The off-diagonal element of the Jones matrix shows the polarization conversion. The subscript of the element contains the information on the polarization of the input light and output light. For example: J_{LR}^R is the RCP input and LCP reflect. Because of the Non-reciprocity, upward and downward reflection and transmission have different polarization conversion efficiency. These properties show us the potential of application in a photonic device. Based on the MO TCMT, we could get the polarization conversion efficiency under forward incidence and backward incidence. From the Jones matrix, we get the polarization conversion efficiency in the form:

$$\zeta_{f,L\to R} = |J_{RL}^{T,f}|^2 = |\frac{d_R^u k_L^d}{\eta}|^2, \tag{S50}$$

$$\zeta_{f,R\to L} = |J_{LR}^{T,f}|^2 = |\frac{d_L^u k_R^d}{\eta}|^2, \tag{S51}$$

$$\zeta_{b,L\to R} = |J_{RL}^{T,b}|^2 = |\frac{d_R^d k_L^u}{\eta}|^2, \tag{S52}$$

$$\zeta_{b,R\to L} = |J_{LR}^{T,b}|^2 = |\frac{d_L^d k_R^u}{\eta}|^2. \tag{S53}$$

9 Far-field polarization and Pancharatnam–Berry phase

We start with a local basis where the polarization direction for upward radiation is $\theta^u = 0°$. Because we need the Stokes parameter to analyze far-field polarization, we write the corresponding Stokes parameter of upward radiation in the form:

$$S_0^\beta = |d_x^\beta|^2 + |d_y^\beta|^2, \tag{S54}$$

$$S_1^\beta = |d_x^\beta|^2 - |d_y^\beta|^2, \tag{S55}$$

$$S_2^\beta = 0, \tag{S56}$$

$$S_3^\beta = 2\sigma^\beta |d_x^\beta||d_y^\beta|, \tag{S57}$$

Here $\sigma^\beta = \pm 1$ is left and right handedness,$\beta = u, d$represent upward and downward respectively. Then the coupling coefficients are

$$d_x^\beta = \sqrt{\frac{S_0^\beta + S_1^\beta}{2}}, \tag{S58}$$

$$d_y^\beta = i\sigma^\beta \sqrt{\frac{S_0^\beta - S_1^\beta}{2}}, \tag{S59}$$

For any arbitrary polarization direction, a rotation matrix $R(\theta)$ can be used to describe the coupling coefficients:

$$R(\theta) = \begin{pmatrix} \cos(\theta) & -\sin(\theta) \\ \sin(\theta) & \cos\theta) \end{pmatrix}. \tag{S60}$$

and the coupling coefficients:

$$\begin{pmatrix} d_x^\beta(\theta^\beta) \\ d_y^\beta(\theta^\beta) \end{pmatrix} = R(\theta) \begin{pmatrix} d_x^\beta(\theta^\beta = 0) \\ d_y^\beta(\theta^\beta = 0) \end{pmatrix}. \tag{S61}$$

That means:

$$d_x^\beta = \sqrt{\frac{S_0^\beta + S_1^\beta}{2}} \cos\theta^\beta - i\sigma^\beta \sqrt{\frac{S_0^\beta - S_1^\beta}{2}} \sin\theta^\beta, \tag{S62}$$

$$d_y^\beta = \sqrt{\frac{S_0^\beta + S_1^\beta}{2}}\sin\theta^\beta + i\sigma^\beta\sqrt{\frac{S_0^\beta - S_1^\beta}{2}}\cos\theta^\beta. \tag{S63}$$

Remember that now we don't discuss under the circular basis, which is more convenient, so there is:

$$d_L^\beta(\theta^\beta) = \frac{1}{\sqrt{2}}[\sqrt{\frac{S_0^\beta + S_1^\beta}{2}}(\cos\theta^\beta + i\sin\theta^\beta) + i\sigma^\beta\sqrt{\frac{S_0^\beta - S_1^\beta}{2}}(-\sin(\theta^\beta) + i\cos(\theta^\beta)]. \tag{S64}$$

By using the Euler formula:

$$d_L^\beta(\theta^\beta) = \frac{e^{i\theta^\beta}}{\sqrt{2}}(\sqrt{\frac{S_0^\beta + S_1^\beta}{2} - \sigma^\beta\sqrt{\frac{S_0^\beta - S_1^\beta}{2}}}). \tag{S65}$$

For convenience, we note that:

$$A^\beta = \sqrt{\frac{S_0^\beta + S_1^\beta}{2}}, \tag{S66}$$

$$B^\beta = \sqrt{\frac{S_0^\beta - S_1^\beta}{2}}. \tag{S67}$$

Similarly:

$$k_R^\beta(\theta^\beta) = \frac{e^{i\theta^\beta}}{\sqrt{2}}(A^\beta + \sigma^\beta B^\beta). \tag{S68}$$

Now we consider one element in the Jones matrix $J_{LR}^T = \frac{1}{\eta}d_L^u k_R^d$ as an example:

$$J_{LR}^T = \frac{1}{2\eta}[e^{i\theta^\beta}(A^u - \sigma^u B^u)][e^{-i\theta^\beta}(A^d + \sigma^d B^d)]. \tag{S69}$$

The phase is:

$$\phi_{LR}^T = \arg(\frac{1}{\eta}) + (\theta^u - \theta^d) + \arg[(A^u - \sigma^u B^u)(A^d + \sigma^d B^d)]. \tag{S70}$$

As the definition of topological charge:

$$l_{LR}^T = \frac{1}{2\pi}\oint_C \nabla_k \phi_{LR}^T d\mathbf{k}. \tag{S71}$$

The first term is about resonance instead of singularity, so it is 0.The second term is about a vortex. The third term is also 0 because there are no phase singularities. But when $\sigma^u \to -\sigma^u$ The third term brings π, inducing an arrow reversal in polarization. But this will not influence the topological charge.

Symmetries	Representation	Possible charges	Eigenvalue of C_2
C_2	A ($C_2 = +1$)	$\pm 1 + 2n$	$+1$
	B ($C_2 = -1$)	$0 + 2n$	-1
C_3	A ($C_3 = +1$)	$1 + 3n$	
C_4	A ($C_4 = +1$)	$1 + 4n$	$+1$
	B ($C_4 = -1$)	$-1 + 4n$	$+1$
C_6	A ($C_6 = +1$)	$1 + 6n$	$+1$
	B ($C_6 = -1$)	$-2 + 6n$	-1

Table 2: Topological charges and eigenvalues under different symmetries[5]

10 The relationship between topological charge and symmetry

The possible charge of at-Γ BIC is determined by the eigenvalue of the rotational symmetry of a system as exhibited in Table 2. Here, $n = 0, \pm 1, \pm 2, ...$ is an integer.

11 Enlarged version of panels in figure 6

Here are enlarged pictures of the panels in figure 6.

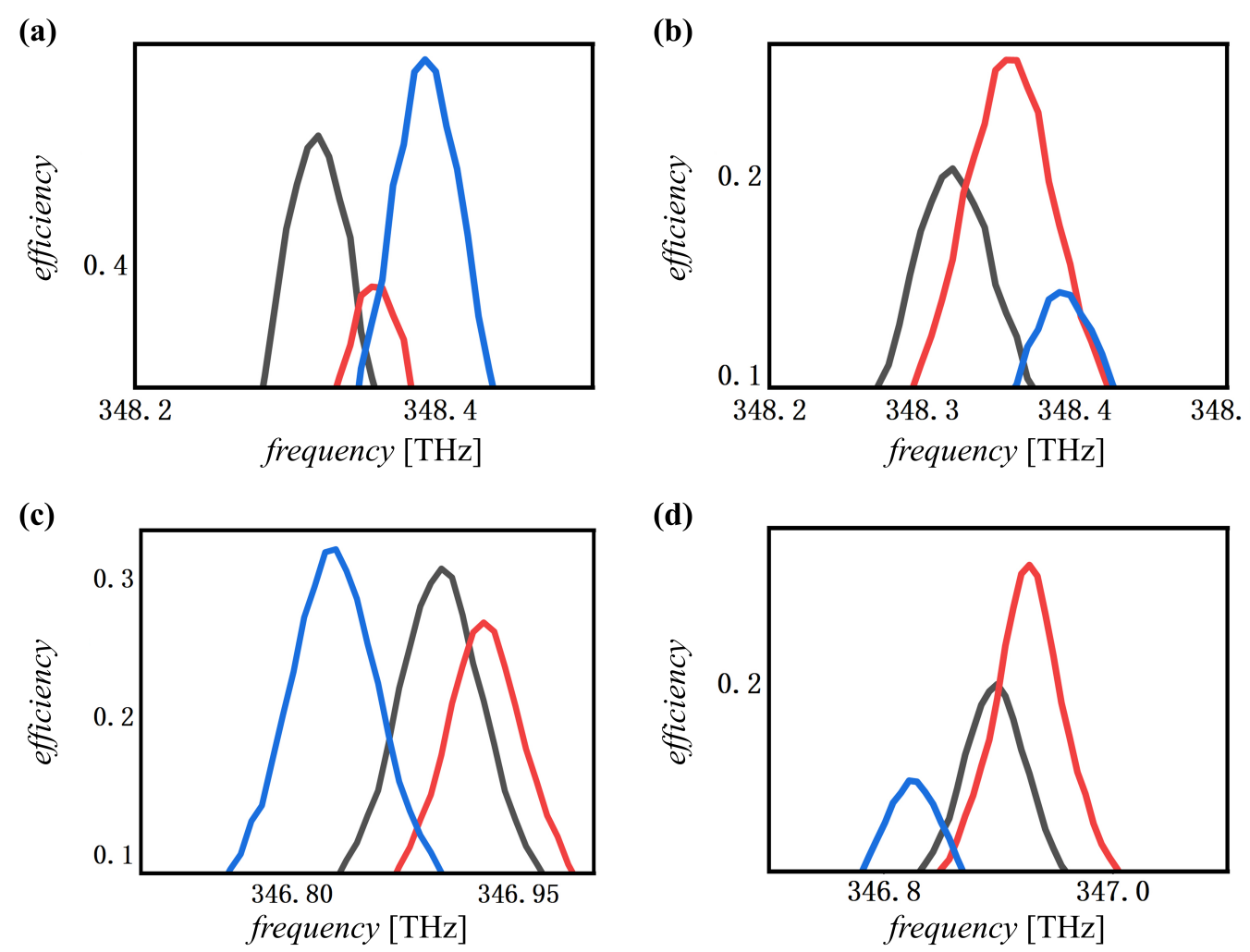

Figure 1: (a)enlarged pictures of (a) in figure 6 (b)enlarged pictures of (b) in figure 6.(c)enlarged pictures of (c) in figure 6.(d)enlarged pictures of (d) in figure 6

References

[1] M. S. Dresselhaus, G. Dresselhaus, and A. Jorio, *Group theory: application to the physics of condensed matter*. Springer Science & Business Media, 2007.

[2] K. J. Buschow, *Handbook of magnetic materials*, vol. 15. Elsevier, 2003.

[3] R. D. V. Meade, S. G. Johnson, and J. N. Winn, "Photonic crystals: Molding the flow of light," 2008.

[4] W. Suh, Z. Wang, and S. Fan, "Temporal coupled-mode theory and the presence of non-orthogonal modes in lossless multimode cavities," *IEEE Journal of Quantum Electronics*, vol. 40, no. 10, pp. 1511–1518, 2004.

[5] T. Yoda and M. Notomi, "Generation and annihilation of topologically protected bound states in the continuum and circularly polarized states by symmetry breaking," *Phys. Rev. Lett.*, vol. 125, no. 5, p. 053902, 2020.
